# Spatiotemporal dynamics and determinants of medical service efficiency in China

**DOI:** 10.1186/s12913-024-11162-1

**Published:** 2024-06-05

**Authors:** Ting Yang, Yiyi Li, Mingzhen Sun, Jingjing Meng

**Affiliations:** 1 School of Health Services Management, Anhui Medical University, Hefei, Anhui 230032 China; 2 Intelligent Interconnected Systems Laboratory of Anhui Province (Hefei University of Technology), Hefei, Anhui 230009 P.R. China

**Keywords:** Medical service efficiency, Spatiotemporal difference, Influencing factors, Epsilon-based measure

## Abstract

**Background:**

Medical service efficiency is an important indicator for measuring the equity of medical services. Therefore, this study primarily focuses on investigating the spatiotemporal domain to explore both spatial and temporal characteristics, as well as influencing factors that affect medical service efficiency across diverse provinces in China.

**Methods:**

The super Epsilon-based Measure (EBM) unexpected model has previously been utilized to quantify energy eco-efficiency, carbon emission efficiency, and green development efficiency. However, limited studies have applied this method to assess the efficiency of healthcare services. Therefore, this study investigates the application of the super-EBM-unexpected model in evaluating medical service efficiency, and further integrates spatial econometric models to explore the influencing factors of medical service efficiency and aims to identify potential avenues for improvement.

**Results:**

The average efficiency of medical services in the 31 provinces of China ranges from 0.6 to 0.7, indicating predominantly low efficiency values. However, economically developed coastal areas exhibit relatively high efficiency levels above 1. Conversely, regions with relatively lower levels of economic development demonstrate lower efficiency rates at approximately 0.3. Evidently, substantial regional disparities exist. For the influencing factors, the enhancement of residents' living standards can effectively foster the medical service efficiency, while residential living standards of nearby areas can also exert an impact in this region. The influence of educational attainment on medical service efficiency exhibits a significant inhibitory effect.

**Conclusions:**

The majority of China's 31 provinces exhibit suboptimal medical service efficiency, with notable regional disparities. Future policy initiatives should be tailored to address the unique challenges faced by regions with lower levels of economic development, prioritizing enhancements in both the efficacy and quality of their healthcare systems.

## Introduction

High-quality medical services serve as a fundamental pillar of human health [1]. Consequently, the healthcare system plays a crucial role in determining the overall well-being of a population [2], with its primary objective being to ensure public health [3]. As the world's largest developing country, China has implemented various strategies and policies in recent years aimed at providing safe, effective, convenient, and affordable medical and healthcare services to its populace. Consequently, healthcare resources have witnessed continuous growth in China while advancements in medical technology capabilities and improvements in medical quality have been consistently observed [4]. Simultaneously, there has been an increasing demand for high-quality medical services due to economic development, rising incomes, and enhanced health literacy among individuals [5]. This surge in demand has resulted in overcrowding within large hospitals while smaller hospitals often receive inadequate attention. The development of China's healthcare industry is characterized by imbalances and insufficiencies leading to an overall low efficiency level of medical services provided [6–8]. Therefore, it is imperative to identify the spatiotemporal characteristics influencing medical service efficiency within China which can aid in formulating more effective public health policies.

Currently, existing studies have investigated medical service efficiency and its influencing factors from various perspectives, using different methods and targeting different research objects. Some studies have evaluated the operational efficiency of hospitals and other decision-making units (DMUs) [9–11]. Other studies take the whole country or a specific region (e.g., a city group, province, or city) as the DMU to evaluate medical service efficiency in different time periods [12–18]. Data Envelopment Analysis (DEA) is the predominant approach employed in reviewing the relevant literature [19–21]. Determining the evaluation index system is the most important step in measuring regional medical service efficiency. When using DEA, appropriate inputs and outputs are essential for meaningful analysis [22]. Previous studies designed different evaluation indicator systems based on different dimensions. However, it is noteworthy that most studies have overlooked the consideration of unexpected outputs when designing evaluation indicator systems. The medical and health service system is a complex entity, necessitating a comprehensive assessment of multiple factors to measure its operational efficiency. Therefore, ensuring holistic integrity within the evaluation index system assumes paramount importance. Moreover, when examining the determinants of service efficiency in medical and healthcare institutions, Tobit models are predominantly employed while spatial effects remain inadequately accounted for [23, 24]. Given individuals' inclination towards superior medical resources, interregional diagnosis and treatment phenomena become conspicuous, thereby underscoring the paramount importance of considering spatial factors.

Therefore, to address the limitations of previous studies, this study incorporates both expected and unexpected outputs in establishing the evaluation index system. Based on this, we apply the super- Epsilon-based Measure (EBM) -unexpected model to evaluate the medical service efficiency of 31 provinces across China. Additionally, a spatial econometric model is employed to analyze spatiotemporal differences and influencing factors of medical service efficiency among different provinces, attempting to identify avenues for improvement.

## Data

This study takes 31 provinces of China as the research object, without considering Hong Kong, Macao, and Taiwan, due to data availability. Referring to existing evaluation systems for medical service efficiency [25], we select investment indicators in three aspects: human, material, and financial resources. Human investment is reflected by the number of technical health personnel per thousand people, material investment is reflected by the number of medical and health institutions in each province, and government emphasis on healthcare is reflected by per capita financial expenditure on healthcare.

The purpose of public health investment is to improve public health by improving the capacity of medical and health services. Therefore, this study selects output indicators from two perspectives: the level of medical and health services and the level of public health. Regarding the level of medical and health services, we select two indicators: the number of outpatient and emergency patients, reflecting the outpatient services of medical institutions, and the number of discharged patients, reflecting the inpatient services of medical institutions. Regarding public health level, the indicators include population mortality, average life expectancy, infant mortality, mortality of children under five years old, and maternal mortality. Based on previous studies and data availability [25], we select the population mortality rate and maternal mortality rate to measure the public health level of residents in the provinces where they live. The higher these two indicators are, the lower the public health level of residents. Therefore, we take these two indicators as unexpected births. Table 1 shows the specific meanings of the indicators.

**Table 1:** Evaluation index system for medical service efficiency in China

Index	Index composition	Indicators
Input index	Financial input	Per capita government expenditure on healthcare (yuan)
	Manpower input	Number of health technicians per 1000 persons (person)
	Material input	Medical and health institution number (unit)
Expected output index	Service volume of medical and health institutions	Number of patients discharged from medical and health institutions (million person times)
	Service volume of medical and health institutions	Number of outpatients and emergency patients in medical and health institutions (100,000 person times)
Unexpected output index	Maternal and child health level	Maternal mortality rate (l/100,000)
	Public health level	Human mortality rate (‰)
Influence factor	Economic development level (PGDP)	Per capita Gross Domestic Product (yuan)
	Urbanization level (Urbanization)	Proportion of urban population in total population (%)
	Educational level (Educational)	Average number of students in school per 100,000 population (higher education) (person)
	Aging degree (Aging)	The proportion of population aged 65 and over (%)
	People living level (Living)	Per capita disposable income (yuan)
	The government's focus on the healthcare sector (Expenditure)	Expenditure for Health Care (100 million yuan)

**Fig. 1:**
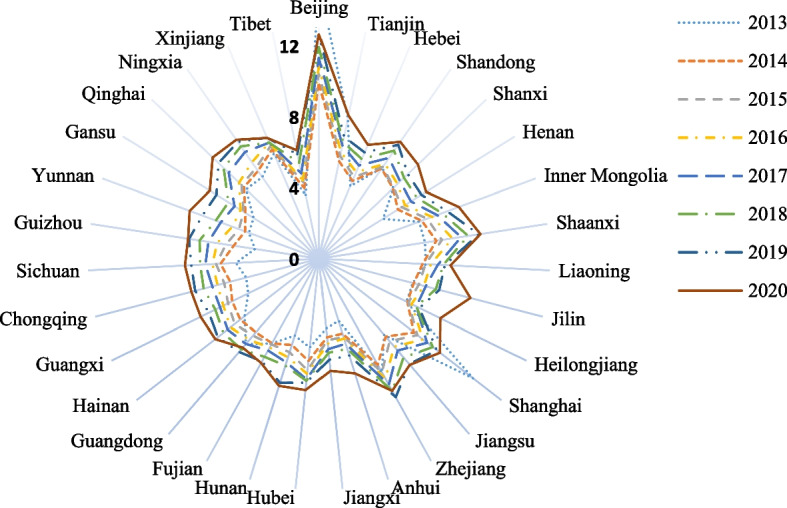
Number of health technicians per 1000 people of 31 provinces from 2013 to 2020

**Fig. 2:**
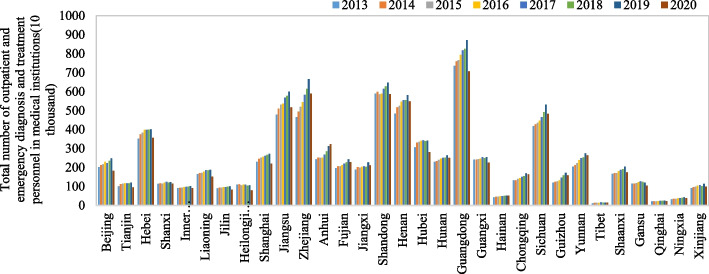
Total number of outpatient and emergency patients in medical institutions of 31 provinces from 2013 to 2020

This study analyzes changes in medical service efficiency in various Chinese provinces. Based on data availability, the selected time range for the panel data is 2013–2020. The data for each variable come from the *China Statistical Yearbook*, *China Social Statistics Yearbook*, *China Health and Health Statistics Yearbook*, and statistical yearbooks of various provinces from 2014 to 2021. Table 2 shows the descriptive statistical results for each variable. To compare the medical resources and medical services of the 31 Chinese provinces, we also create a radar chart of the number of health technicians per thousand people in the 31 provinces and the total number of outpatient and emergency medical institutions (Figs. 1 and 2).

**Table 2:** Descriptive statistics of the variables

Variables	Mean	Standard deviation	Minimum	Maximum
Per capita government expenditure on healthcare	1123.63	500.71	469.74	3955.34
Number of health technicians per 1000 persons	6.53	1.52	3.64	15.46
Medical and health institution number	32,005.38	22,384.16	4321.00	86,939.00
Number of patients discharged from medical and health institutions	245.13	191.72	11.13	871.97
Number of outpatients and emergency patients in medical and health institutions	73.48	50.16	1.99	201.32
Maternal mortality rate	15.63	16.71	1.10	154.50
Human mortality rate	6.18	0.93	4.26	11.50
Economic development level (PGDP)	59,221.28	27,744.64	22,922.00	164,889.00
Urbanization level (Urbanization)	58.51	12.58	23.71	89.60
Educational level (Educational)	2656.44	788.34	1133.00	5534.00
Aging degree (Aging)	10.80	2.56	4.98	17.42
People living level (Living)	25,113.13	11,234.84	9746.80	72,232.00
The government's focus on the healthcare sector (Expenditure)	436.59	273.34	40.29	1772.99

As shown in Fig. 1, from 2013 to 2020, the number of health technicians per 1000 people in Chinese provinces shows an increasing trend year by year, except in Beijing and Shanghai, most notably in Guizhou, Yunnan, Anhui, and other provinces. Developed areas such as Beijing and Shanghai have rich medical resources, but in recent years this difference has been narrowing. We can see in Fig. 2 that from 2013 to 2019, the total number of outpatient and emergency treatment personnel in medical institutions in the 31 provinces is increasing year by year. In 2020, however, affected by COVID-19, this number decreased significantly. We also find that the total number of outpatient and emergency patients in medical institutions in Guangdong, Zhejiang, Jiangsu, Shandong, Henan, and other provinces is significantly higher than that in other provinces, which is related to the large resident populations in these provinces.

In summary, from 2013 to 2020, the total amount of medical resources and medical services in the 31 Chinese provinces shows an upward trend. It should also be noted, however, that there are large differences in the total amount of medical resources and medical services in each province, which is closely related to the geographical location, population, development level, ecological conditions, and other factors of different provinces. Regarding the medical service efficiency situation in each province and the differences between them, we use the super-EBM-unexpected model, spatial econometric model, and other methods to compare and analyze the spatial and temporal differences in medical service efficiency and their effect mechanisms at the provincial level.

## Methods

### Super-EBM-unexpected model

DEA was proposed by Charnes et al. in 1978 and has been widely used since then [26]. In the realm of traditional DEA methods, the Charnes, Cooper, and Rhodes (CCR) model and the Banker, Charnes, and Cooper (BCC) model stand out as the two most extensively employed models. The DEA method offers advantages in mitigating subjective factors, streamlining operations, and minimizing errors [27]. However, certain issues persist, leading to the development of numerous novel approaches. Addressing measurement errors resulting from the neglect of relaxation variables in traditional CCR and BCC models, Thorn introduced the Slacks-based Measure (SBM) model for efficiency measurement based on non-radial and non-angular relaxation variables in 2001 [28]. Building upon this foundation, Tone and Tsutsui further proposed the Epsilon-based Measure (EBM) model by considering the radial ratio between input frontier value and actual value [29]. It not only considers the radial ratio between the target value and the actual value but also reflects the differential nonradial relaxation variables between inputs, finds the gap between the target value and the actual value, and improves the accuracy of results [30]. However, it has rarely been used in healthcare-related research. Moreover, conventional DEA models fail to effectively differentiate multiple efficient decision-making units. Therefore, Anderson and Peterson proposed an enhanced super-efficiency DEA model [31]. In this study, we integrate the EBM model with super efficiency model and introduces the concept of unexpected outputs to establish a novel evaluation framework, known as the super-EBM-unexpected model, for revising the assessment of medical service efficiency across 31 provinces in China.This super-EBM-unexpected model has previously been employed to quantify energy eco-efficiency, carbon emission efficiency, and green development efficiency [32,33].

The nondirected super-EBM-unexpected model is described as Eqs. (1, 2, 3, 4 and 5).

1 $${r}^{*}=min\frac{\theta -{\epsilon }_{x}\sum_{i=1}^{m}\frac{{{w}_{i}^{-}s}_{i}^{-}}{{x}_{i0}}}{\varphi +{\epsilon }_{y}\sum_{r=1}^{s}\frac{{{w}_{r}^{+}s}_{r}^{+}}{{y}_{r0}}+{\epsilon }_{z}\sum_{p=1}^{q}\frac{{{w}_{p}^{z-}s}_{p}^{z-}}{{z}_{p0}}}$$

2 $$\sum_{j=1}^{n}\limits{x}_{ij}{\lambda }_{j}+{s}_{i}^{-}=\theta {x}_{i0} \left(i=\text{1,2},\cdots ,m\right)$$

3 $$\sum_{j=1}^{n}\limits{y}_{rj}{\lambda }_{j}-{s}_{r}^{+}=\varphi {y}_{r0} \left(r=\text{1,2},\cdots ,s\right)$$

4 $$\sum_{j=1}^{n}\limits{z}_{pj}{\lambda }_{j}+{S}_{p}^{-}=\varphi {z}_{p0} \left(p=\text{1,2},\cdots ,q\right)$$

5 $${\lambda }_{j}\ge 0, {s}_{i}^{-},{s}_{r}^{+},{S}_{p}^{-}\ge 0$$

where ${r}^{*}$ denotes medical service efficiency. The meanings of other indicators can be found in Yang et al. [34]. The objective function of Eq. (1) is supplemented by constraints from Eqs. (2, 3, 4 and 5).

### Spatial autocorrelation analysis

This study uses spatial autocorrelation to analyze the spatial correlations of medical service efficiency among the selected regions. We use the single-variable global spatial autocorrelation test to measure the spatial correlation degree of the attribute values in the whole region, measured by Moran’s *I*. The Moran's I statistic typically ranges between -1 and 1, where positive values indicate a positive spatial correlation, negative values indicate a negative spatial correlation, and zero indicates no spatial correlation.

To determine spatial correlations between medical service efficiency and economic development level, this study further uses the bivariate global Moran’s index to identify the effect of the economic development of the 31 provinces on medical service efficiency in surrounding provinces.

### Spatial metrological model

This study mainly uses a super-EBM-unexpected model to measure the operational efficiency of medical services in China’s various regions while also studying the spatial effects and influencing factors using a spatial econometric model. Since medical service efficiency might have spatial effects, and economic development, urbanization, education, aging, and other factors in adjacent areas might affect medical service efficiency in a region, we construct a spatial econometric model—mainly establishing a spatial Durbin model (SDM)—to analyze the factors affecting medical service efficiency. The specific model is as follows:

6 $$ln{Y}_{it}={\beta }_{0}+\gamma W*ln{Y}_{it}+{\beta }_{1}ln{PGDP}_{it}+{\beta }_{2}ln{Educational}_{it}+{\beta }_{3}ln{Urbanization}_{it}+{\beta }_{4}ln{Aging}_{it}+{\beta }_{5}ln{Living}_{it}+{\beta }_{6}ln{Expenditure}_{it}+{\rho }_{1}W*ln{PGDP}_{it}+{\rho }_{2}W*\text{l}n{Educational}_{it}+{\rho }_{3}W*ln{Urbanization}_{it}+{\rho }_{4}W*ln{Aging}_{it}+{\rho }_{5}W*ln{Living}_{it}+{\rho }_{6}W*ln{Expenditure}_{it}+{\varepsilon }_{it}$$

where ${Y}_{it}$ denotes medical service efficiency; ${PGDP}_{it}$ is the per capita Gross Domestic Product (GDP) of region *i* in year *t*, representing the level of economic development; ${Educational}_{it}$ is the average number of students in school (higher education) per 100,000 people in the *i*th region in year *t*, representing the level of education; ${UL}_{it}$ is the proportion of the urban population in the *i*th region in the total population in year *t*, reflecting the urbanization level of the region; and ${Aging}_{it}$ is the proportion of the population aged 65 years and above in the total population of region *i* in year *t*, reflecting the degree of population aging; ${Living}_{it}$ is the per capita disposable income of residents in *i*th region in year *t*, reflecting residents’ living standards. The larger the indicator, the greater the degree of aging in the region. ${Expenditure}_{it}$ represents the health expenditure of the i-th region in the t-th year of the government's general public budget allocation, serving as an indicator of the government's prioritization of investments in the healthcare industry.$W*{Y}_{it}$ represents the spatial term of the interpreted variable, ${W}_{ij}{*X}_{jtk}$ is the spatial term of the influencing factor variable, and *W* represents the spatial weight matrix; we use the adjacent spatial weight matrix in this study. ${\varepsilon }_{it}$ is the random error term.

## Results

### Temporal and spatial evolution characteristics

**Fig. 3:**
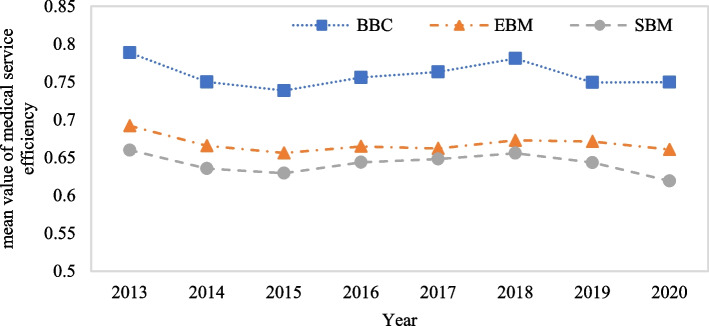
Changes in the mean value of medical service efficiency in China from 2013 to 2020

To test and verify the effectiveness of the EBM model, we first use MaxDEA 8.0 to comparatively analyze the calculation results of the three DEA models (BCC, SBM, and EBM), and all three models involve unexpected outputs. Figure 3 shows the results.

**Fig. 4:**
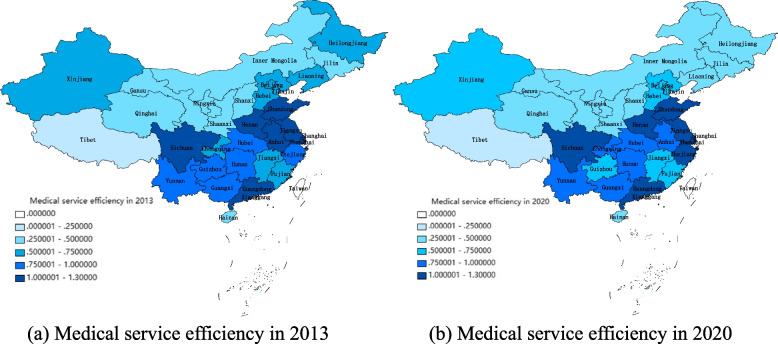
Spatial pattern of medical service efficiency in China

We can see from Fig. 3 that the estimation results of the super-EBM-unexpected model considering unexpected output is between that of the SBM model and the BCC model. This reflects the fact that the EBM model overcomes the shortcomings of SBM and BCC model and improves the accuracy of the estimation result. Therefore, we select the EBM model containing unexpected output to estimate medical service efficiency in the 31 provinces. Table 3 shows the results, and as shown in Fig. 4, a spatial pattern chart is drawn by selecting 2013 and 2020 data. We can also see in Fig. 3 that the average medical service efficiency of the 31 provinces is between 0.6 and 0.7, meaning the efficiency value is low. Specifically, from 2013 to 2015, there is a downward trend, and from 2016 to 2019, there is a slow upward trend. In 2020, however, there is a downward trend. This is related to increased attention healthcare in recent years as well as the effects of COVID-19.

**Table 3:** Medical service efficiency in Chinese provinces, 2013–2020

Region	DMU	2013	2014	2015	2016	2017	2018	2019	2020
Northern coastal area	Beijing	0.5596	0.5635	0.5628	0.6450	0.6067	0.6191	0.6300	0.6691
	Tianjin	0.5381	0.5150	0.5001	0.5093	0.5508	0.5521	0.5592	0.5625
	Hebei	0.6916	0.7347	0.7171	0.7548	0.7268	0.6930	0.6767	0.6156
	Shandong	1.0188	1.0196	1.0170	1.0147	1.0294	1.0218	1.0169	1.0277
Middle reaches of the Yellow River	Shanxi	0.3157	0.3113	0.2910	0.3195	0.3190	0.3318	0.3276	0.2987
	Inner Mongolia	0.3327	0.3302	0.3018	0.3128	0.3189	0.3369	0.3175	0.2945
	Henan	1.0352	1.0087	1.0098	1.0077	1.0065	1.0895	1.0373	1.1117
	Shaanxi	0.4620	0.4427	0.4430	0.4581	0.4709	0.5106	0.5179	0.4962
Northeast China	Liaoning	0.5465	0.5577	0.5816	0.5915	0.5819	0.5749	0.5498	0.4840
	Jilin	0.4147	0.4303	0.3981	0.4005	0.3936	0.3854	0.3926	0.2963
	Heilongjiang	0.6160	0.6048	0.6121	0.6427	0.6420	0.6298	0.6469	0.4320
Eastern coastal area	Shanghai	1.1878	1.1597	1.1709	1.2037	1.1465	1.1528	1.1038	1.0968
	Jiangsu	1.0294	1.0227	1.0282	1.0317	1.0313	1.0253	1.0208	1.0301
	Zhejiang	0.9051	0.9193	0.9341	0.9639	1.0182	1.0765	1.0165	1.0281
Middle reaches of Changjiang River	Anhui	1.0060	0.9042	0.8831	0.9114	0.9212	0.9283	0.8934	0.8300
	Jiangxi	0.7234	0.6026	0.6011	0.5991	0.6337	0.6585	0.6411	0.6435
	Hubei	0.9244	0.8810	0.8419	0.8586	0.8598	0.9247	0.9490	0.7619
	Hunan	0.9225	0.9037	0.9240	0.9313	0.9273	0.9344	0.9147	0.9334
Southern coastal area	Fujian	0.5422	0.5159	0.4788	0.4646	0.4479	0.4668	0.4894	0.5225
	Guangdong	1.2682	1.3663	1.3803	1.3160	1.2035	1.1848	1.1486	1.1882
	Hainan	0.3870	0.3273	0.3339	0.3282	0.3240	0.3154	0.3122	0.3198
Southwest China	Guangxi	0.8559	0.7098	0.6778	0.6617	0.6520	0.6746	0.7367	0.7707
	Chongqing	0.7316	0.7479	0.7150	0.7124	0.7355	0.7294	0.7593	0.7891
	Sichuan	1.0123	0.9805	0.9758	0.9762	1.0008	0.9144	0.9152	1.0005
	Guizhou	0.7953	0.6135	0.5668	0.5570	0.5707	0.6359	0.6550	0.6800
	Yunnan	0.8164	0.8087	0.7812	0.7993	0.7837	0.8436	0.8599	0.9067
Northwestern China	Gansu	0.3710	0.3330	0.3312	0.3542	0.3523	0.3938	0.4273	0.4113
	Qinghai	0.2912	0.2533	0.2277	0.2319	0.2263	0.2179	0.2183	0.2574
	Ningxia	0.4252	0.3537	0.3366	0.3516	0.3527	0.3471	0.3466	0.3667
	Xinjiang	0.6539	0.6500	0.6396	0.6278	0.6152	0.6180	0.6662	0.5722
Tibet		0.0722	0.0698	0.0808	0.0757	0.0803	0.0718	0.0667	0.0864
Number of DMUs with efficiency value greater than 1		7	4	5	5	7	6	6	7
Number of DMUs with efficiency less than 0.6		13	13	14	14	13	12	12	14

**Fig. 5:**
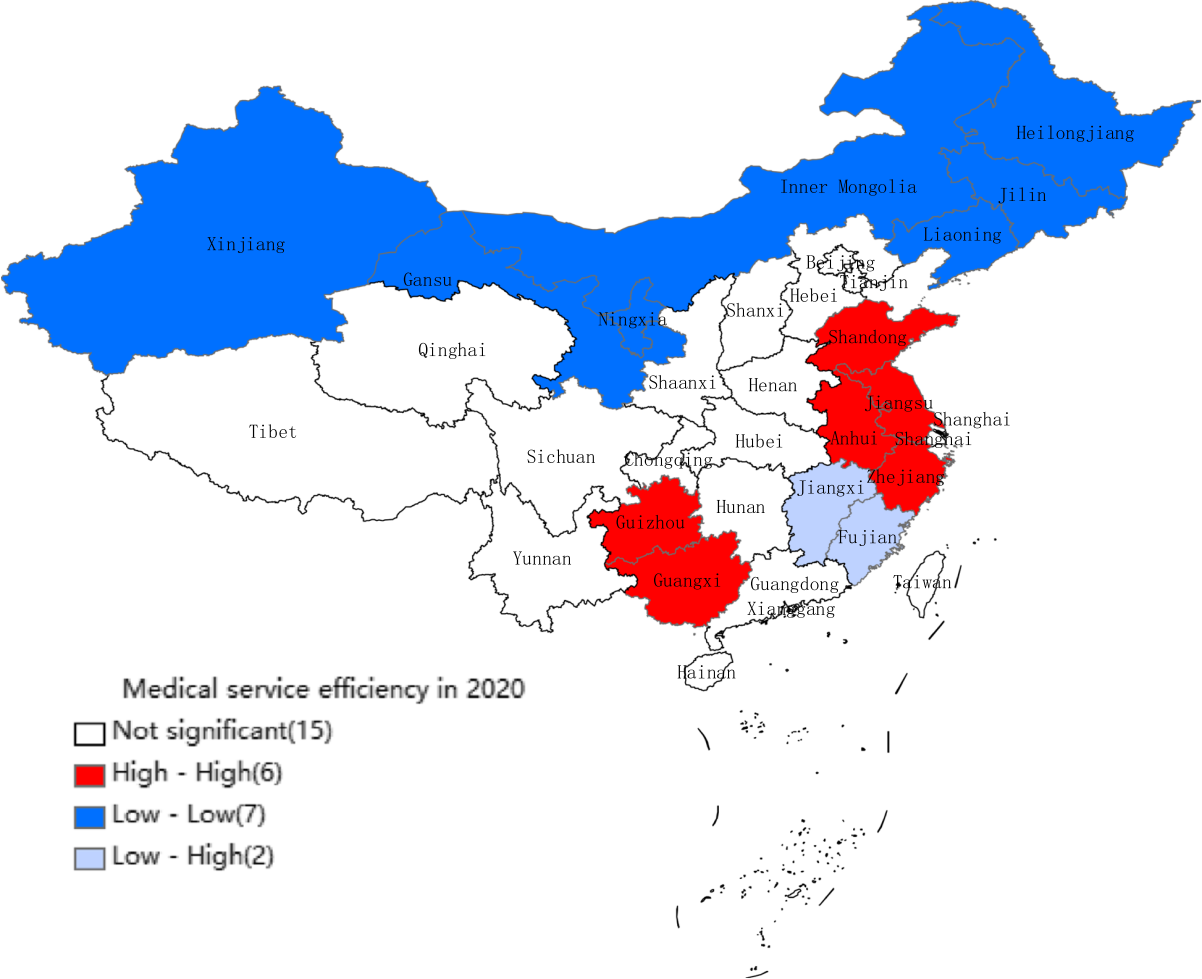
LISA cluster map of medical service efficiency in China in 2020

We can see in Table 3 and Fig. 4 that medical service efficiency in coastal areas such as Guangdong, Shanghai, Jiangsu, and Shandong is high, and fluctuations in medical service efficiency in each province from 2013 to 2020 are small. In addition, the number of DMUs with efficiency values greater than 1 increases from five in 2016 to seven in 2020, and the number of DMUs with efficiency values less than 0.6 decreases from 14 in 2016 to 12 in 2019, but there is a rebound trend in 2020. In other words, the distribution of medical service efficiency in China is high in the coastal areas and low in inland areas. Medical service efficiency is relatively high in provinces with a high level of economic development. In order to provide a more intuitive illustration of this phenomenon, the present study computes the local Moran index based on the efficiency of medical services in 31 provinces across China during 2020. Subsequently, Local Indicators of Spatial Association (LISA) clustering maps are generated for regions that have passed significance tests, facilitating an analysis of the localized spatial clustering phenomenon pertaining to medical service efficiency, as shown in Fig. 5.

As shown in Fig. 5, the regions with higher medical service efficiency are clustered together, and those with lower medical service efficiency are clustered together as well. The clustered areas with higher medical service efficiency are mainly distributed in the eastern coastal areas, especially in Shanghai, Jiangsu, Shandong, and Anhui, where economic and social development levels are relatively high. The areas with lower medical service efficiency are mainly clustered in the northwest and northeast, such as Xinjiang, Liaoning, Inner Mongolia, Heilongjiang, and Gansu. These areas have low levels of economic development.

### Regional difference analysis

**Fig. 6:**
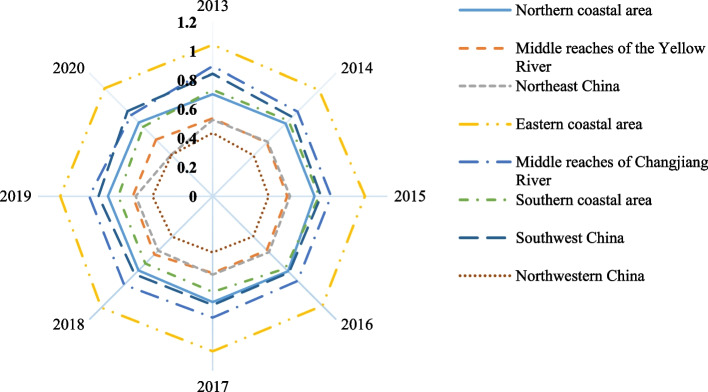
Temporal evolution of medical service efficiency in eight regions of China from 2013 to 2020

To further examine differences in regional medical service efficiency, following traditional administrative divisions, 30 provinces (Xinjiang is analyzed as a single element since it has the lowest medical service efficiency and is significantly different from other provinces) are divided into the northern coastal areas, northeast regions, eastern coastal areas, southern coastal areas, middle reaches of the Yellow River, middle reaches of the Yangtze River, southwest regions, and greater northwest regions. Figure 6 shows the medical service efficiency of the eight regions from 2013 to 2020.

**Fig. 7:**
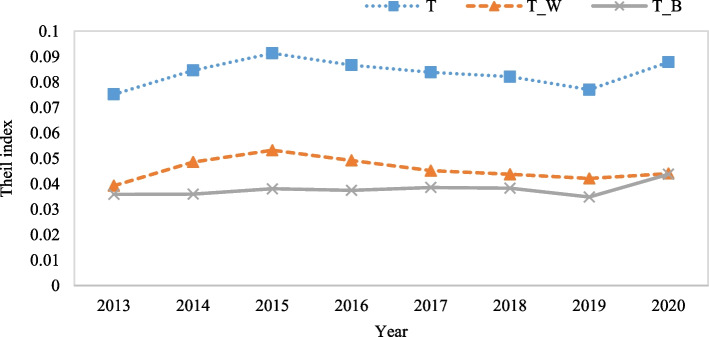
Theil index of medical service efficiency in eight regions of China

Overall, while medical service efficiency fluctuates slightly in the eight regions, the regional differences are obvious. Specifically, the eastern coastal area exhibits the highest efficiency in medical services; since 2013, its efficiency value has consistently exceeded 1, indicating effectiveness. The middle reaches of the Yangtze River and the southwest region also demonstrate high medical service efficiency, approaching a value of 1 with a gradual upward trend. Conversely, regions such as the greater northwest, northeast, and middle reaches of the Yellow River exhibit inefficiency with an efficiency value below 0.6. Notably, medical service efficiency in the greater northwest is found to be significantly lower compared to other regions in China. To further analyze the degrees of difference in medical service efficiency between these eight regions, we use the Thiel index to examine the specific causes of medical service efficiency. Figure 7 shows the results.

In Figs. 3 and 7, we can see that medical service efficiency in the eight regions shows an inverted N-type change feature from 2013 to 2020, while the Thiel index also shows a similar N-type change, which can be roughly divided into three stages. In the first stage (2013–2015), medical service efficiency decreases, and the Theil index rises from 0.07522 in 2013 to 0.09135 in 2015, expanding regional differences. In the second stage (2015–2019), medical service efficiency increases, but the Theil index shows a downward trend, and the degree of regional difference gradually eases. In the third stage (2019–2020), medical service efficiency shows a downward trend, the volatility of the Thiel index increases sharply, and regional differences widen. Further combining the decomposition of the Theil index, we can see that intra- and intergroup differences are the sources of differences in regional medical service efficiency, and the intragroup differences are slightly greater than the intergroup differences.

### Spatial autocorrelation test

To explore whether there is spatial correlation between medical service efficiency, economic development level, and other relevant indicators in the 31 Chinese provinces, we use spatial autocorrelation for analysis, GeoDa to establish a spatial weight adjacency matrix, and single-variable global Moran’s *I* and bivariate Moran’s *I* to test the spatial correlation between medical service efficiency and PGDP in the 31 provinces. Table 4 shows the results.

**Table 4:** Spatial autocorrelation test results for the variables

Year	Medical service efficiency	Per capita GDP	Per capita GDP and medical service efficiency
2013	0.3960^c^	0.4290^c^	0.0950
2014	0.3080^c^	0.4120^c^	0.1460^a^
2015	0.2980^c^	0.4120^c^	0.1840^a^
2016	0.2970^b^	0.4260^c^	0.2530^b^
2017	0.3280^c^	0.4510^c^	0.2830^c^
2018	0.3670^c^	0.4430^c^	0.2840^c^
2019	0.3580^c^	0.3700^c^	0.3160^c^
2020	0.4010^c^	0.3740^c^	0.2950^c^

^a,^ ^b^ and ^c^ denote significance levels at 5%, 1%, and 0.1% respectively

We can see in Table 4 that during the study period, Moran’s *I* values of medical service efficiency in different years are positive (between 0.297 and 0.401) and pass the spatial autocorrelation test at the significance level of 1%. This indicates that medical service efficiency in the 31 Chinese provinces has spatial correlation and shows significant positive correlations in spatial distribution; that is, high-efficiency values coexist with high-efficiency values, and low-efficiency values coexist with low-efficiency values. Therefore, when analyzing the factors influencing medical service efficiency, it is necessary to consider the spatial factors and establish a spatial metrological model. We can also see that since 2016, Moran’s *I* value of medical service efficiency has shown an increasing trend, indicating that the positive spatial characteristics of medical service efficiency in the 31 provinces are becoming stronger. Moran’s *I* of economic development level is also significantly positive, indicating that the economic development level of the 31 provinces also has a positive spatial correlation.

The bivariate Moran’s *I* test can effectively identify the effect of each province’s economic development level on the level of medical service in adjacent areas. Therefore, we conducted further analysis on the spatial correlation between the level of economic development and medical service efficiency in 31 Chinese provinces from 2013 to 2020. The per capita gross domestic product (PGDP) of each province was considered as the independent variable, while medical service efficiency served as the dependent variable (Table 4). Table 4 shows that the bivariate Moran’s *I* values are all positive and mostly pass the significance test, indicating a positive spatial correlation between economic development level and medical service efficiency in the 31 provinces over the years. That is, the economic development level of a region will have a positive effect on medical service efficiency in adjacent regions.

### Factors influencing medical service efficiency

The correlation coefficients between medical service efficiency and various influencing factors were analyzed using Pearson correlation analysis (Table 5). The results presented in Table 5 demonstrate a significant positive correlation between medical service efficiency and the diverse influencing factors, with statistical significance observed at a level of no less than 0.1%.

**Table 5:** Pearson correlation between medical service efficiency and influence factors

Variables	Medical service efficiency	Per capita GDP	Educational	Aging	Urbanization	Living	Expenditure
Medical service efficiency	1						
Per capita GDP	0.3048^c^	1					
Educational	0.1621^b^	0.6907^c^	1				
Aging	0.4266^c^	0.3952^c^	0.4795^c^	1			
Urbanization	0.3049^c^	0.8643^c^	0.7511^c^	0.4624^c^	1		
Living	0.3469^c^	0.9317^c^	0.6684^c^	0.4528^c^	0.8579^c^	1	
Expenditure	0.7315^c^	0.2604^c^	0.1580^a^	0.4106^c^	0.1816^b^	0.3003^c^	1

^a, b^ and ^c^ denote significance levels at 5%, 1%, and 0.1% respectively

Then, the Hausman test is employed to determine whether a fixed effects or random effects model should be used. If the test yields a positive result with a *P*-value less than 0.05, then a fixed effects model is selected; if the test yields a positive result but with a *P*-value greater than 0.05, then a random effects model is chosen; and if the test yields negative results, then a fixed effects model is preferred. Additionally, to demonstrate the efficacy of the spatial Durbin model, we conducted a comparative analysis with the static linear model. Furthermore, we computed both direct and indirect effects for each variable, as presented in Table 6.

**Table 6:** Analysis results of the factors influencing medical service efficiency

	Static linear	SDM	SDM		
			Direct effect	Indirect effect	Total effect
lnPGDP	0.1657^b^ (0.0536)	0.1296^a^ (0.0539)	0.1298^a^ (0.0538)	0.0913 (0.1017)	0.2212^a^ (0.1105)
lnEducational	-0.2086^b^ (0.0711)	-0.1905^b^ (0.0732)	-0.1907^b^ (0.0731)	-0.0530 (0.1110)	-0.2437^a^ (0.1202)
lnAging	0.0019 (0.0642)	0.0305 (0.0702)	0.0311 (0.0703)	0.2185^a^ (0.1046)	0.2496^a^ (0.1120)
lnUrbanization	0.2015 (0.1752)	0.0937 (0.1950)	0.0928 (0.1934)	-0.3032 (0.3132)	-0.2104 (0.3276)
lnLiving	0.3019^c^ (0.0948)	0.4036^c^ (0.1108)	0.4032^c^ (0.1109)	-0.1458 (0.1368)	0.2575^a^ (0.1393)
lnExpenditure	-0.3370^c^ (0.0948)	-0.3149^c^ (0.0531)	-0.3150^c^ (0.0531)	-0.0316 (0.0652)	-0.3466^c^ (0.0722)
${\beta }_{0}$	0.0616^c^ (0.0030)	0.0601^c^ (0.0029)	_	_	_
W*lnPGDP	_	0.1030 (0.1149)	_	_	_
W*lnEducational	_	-0.0588 (0.1303)	_	_	_
W*lnAging	_	0.2490^a^ (0.1221)	_	_	_
W*lnUrbanization	_	-0.3468 (0.3568)	_	_	_
W*lnLiving	_	-0.1700 (0.1600)	_	_	_
W*lnExpenditure	_	-0.0332 (0.0832)	_	_	_
γ	_	0.0091 (0.0981)	_	_	_
R^2^	0.2284	0.2028	_	_	_
Hausman chi2 (*P* value)	22.9 (0.0008)	-10.61	_	_	_

^a, b ^and ^c^ denote significance levels at 5%, 1%, and 0.1% respectively. The numbers in brackets indicate robust standard error, and "-" indicates that they are not included. IF stands for influencing factors

According to the test results of the spatial Durbin model, it is evident that the enhancement of economic development and residents' living standards effectively facilitates the improvement of medical service efficiency. The impact of education level on medical service efficiency is notably negative, possibly due to an increased awareness among individuals with higher education levels regarding their own health concerns, leading to a constant rise in demand for medical care, particularly for high-quality healthcare resources. Consequently, this has resulted in overcrowding in major hospitals and limited access to basic medical institutions, causing a significant imbalance in the healthcare system and subsequently impeding the efficiency of medical services. Furthermore, a negative correlation exists between the allocation of medical and health expenditure in the government's general budget and the efficiency of medical services, highlighting the need to enhance investment strategies in healthcare and foster technological innovation for optimizing operational efficiency within the healthcare system. The urbanization level and population aging do not exhibit any substantial influence on medical service efficiency.

From the perspective of the coefficient of spatial lag term, it is observed that an increasing aging population in neighboring regions exerts a positive influence on the enhancement of medical service efficiency within this region. This finding implies a significant inter-regional phenomenon pertaining to medical treatment. The results align with the estimates and significance levels of the coefficients in the spatial Durbin model, both in terms of direct effects and total effects. The increase in the aging population of neighboring cities has a significant indirect effect on the efficacy of medical services within this region.

## Discussion

The super-EBM-unexpected model was employed to assess the medical service efficiency across 31 provinces in China. The results showed the average efficiency of medical services in 31 provinces of China has ranged from 0.6 to 0.7 in recent years, indicating predominantly low efficiency values and an overall ineffective stage. Despite a gradual upward trend observed in recent years, progress has been sluggish, highlighting the existing scope for enhancing medical efficiency.

Due to disparities in regional economic development and inadequate distribution of medical resources, there exist substantial variations in the efficiency of medical services across different regions, exhibiting a distinct spatial pattern characterized by higher levels along coastal areas and lower levels inland. These significant regional disparities necessitate each province to formulate tailored policies and regulations based on their unique development status, conditions, positioning, and goals to achieve comprehensive and balanced development within the medical and health industry.

The high-quality development of the economy, coupled with the increasing income of residents and the continuous enhancement of national health literacy, has led to a growing demand for superior medical resources and services among individuals. However, in economically underdeveloped regions, the scarcity and inequitable distribution of high-quality medical resources pose significant challenges in meeting the increasingly diverse, high-standard, and personalized healthcare demands of the population. Consequently, there is a notable trend of residents seeking medical treatment across different geographical areas. With the advancement of education and the emergence of an aging population, there has been a growing demand for high-quality medical services. Therefore, in the future, it is imperative for regions to enhance cooperation and exchange, facilitate collaborative construction and sharing of medical resources, enhance the accessibility of medical resources, particularly those of high quality, and ensure equitable access to superior medical and healthcare services for all individuals.

### Strengths and limitations

A strength of this study is that it considers both expected and non-expected outputs when establishing the evaluation index system, thus improving the accuracy of medical service efficiency assessment. Building upon this foundation, the super-EBM-unexpected model is first used in assessing medical service efficiency, objectively presenting their effectiveness across 31 provinces in China. Subsequently, employing a spatiotemporal perspective, the spatial econometric model is used to explore the influencing factors of medical service efficiency.

This study also has certain limitations. Firstly, due to data availability, the scope of relevant data is not comprehensive. Based on more comprehensive data, the evaluation indicators can also be further expanded. Further discussion on the disparities between urban and rural areas can be facilitated by obtaining pertinent index data for both regions in China. Additionally, further analysis of spatial differences could enhance the examination of medical service efficiency convergence.

## Conclusion

The present study is grounded in the field of temporal and spatial analysis, wherein an evaluation index system encompassing expected and unexpected outputs is established. By employing the super-EBM-unexpected model and spatial econometric model, this study examines the temporal and spatial similarities and differences in the medical service efficiency across 31 provinces in China. The research findings indicate that the medical service efficiency in the majority of Chinese provinces is comparatively suboptimal. Although there has been a gradual upward trend in recent years, it remains sluggish, leaving ample room for improvement in medical service efficiency. The regional disparities are evident, demonstrating a distribution pattern characterized by higher levels along the coast and lower levels inland. In the future, increased investments in medical and health resources remains imperative, accompanied by tailored policies aimed at enhancing the efficiency and quality of healthcare systems in economically less developed regions. Moreover, it is crucial to strengthen regional cooperation and exchanges to facilitate collaborative construction and sharing of medical resources, thereby synergistically improving the overall efficiency of the national healthcare system. The findings have implications for the accurate and comprehensive evaluation of regional differences in medical service efficiency and for promoting coordinated improvements in medical service efficiency among different regions in China.

## Data Availability

No datasets were generated or analysed during the current study.
